# Effects and Mechanisms of Electroacupuncture on Chronic Inflammatory Pain and Depression Comorbidity in Mice

**DOI:** 10.1155/2020/4951591

**Published:** 2020-05-28

**Authors:** Hung-Yu Huang, Hsien-Yin Liao, Yi-Wen Lin

**Affiliations:** ^1^Department of Neurology, China Medical University Hospital, Taichung 40447, Taiwan; ^2^College of Chinese Medicine, School of Post-Baccalaureate Chinese Medicine, China Medical University, Taichung 40402, Taiwan; ^3^Department of Acupuncture, China Medical University Hospital, Taichung 40402, Taiwan; ^4^College of Chinese Medicine, Graduate Institute of Acupuncture Science, China Medical University, Taichung 40402, Taiwan; ^5^Chinese Medicine Research Center, China Medical University, Taichung 40402, Taiwan

## Abstract

Comorbidity of chronic pain and major depression disorder (MDD) are common diseases. However, the mechanisms of electroacupuncture (EA) and the responses of N-methyl-D-aspartate receptors in the brain remain unclear. Three injections of complete Freund's adjuvant (CFA) were administered to induce chronic inflammatory pain (CIP). EA was then performed once every other day from days 14 to 28. Behavior tests of chronic pain and depression were evaluated to make sure of the successful induction of comorbidity. We used Western blotting to analyze brain tissue from the prefrontal cortex (PFC), hippocampus, and hypothalamus for levels of phosphorylated N-methyl-D-aspartate receptor subunit 1 (pNR1), NR1, pNR2B, NR2B, and calcium/calmodulin-dependent protein kinase type II alpha isoform (pCaMKII*α*). The mechanical hyperalgesia, thermal hyperalgesia, and depression were observed in the CIP group. Furthermore, decreased levels of N-methyl-D-aspartate receptors (NMDARs) were also noted. Not Sham EA but EA reversed chronic pain and depression as well as the decreased levels of NMDA in the signaling pathway. The CFA injections successfully induced a significant comorbidity model. EA treated the comorbidity by upregulating the NMDA signaling pathway in the PFC, hippocampus, and hypothalamus. Our results indicated significant mechanisms of comorbidity of chronic pain and MDD and EA-analgesia that involves the regulation of the NMDAR signaling pathway. These findings may be relevant to the evaluation and treatment of comorbidity of chronic pain and MDD.

## 1. Introduction

Chronic pain and major depression disorder are common diseases among medical outpatients [1, 2]. In 2010, the economic burdens of MDD and pain in the United States were $210.5 billion [3] and $300 billion [4], respectively. Depression and pain had an estimated cooccurrence of up to 80% in 2015 [5]. Pain adversely affects the prognosis of depression and vice versa. One study indicated that patients with chronic pain had a significantly higher chance of developing depression (30%–54%) than the general population (5%–8%) [6]. Furthermore, patients with chronic pain from physical conditions have longer depressive moods [7]. Similarly, another study showed that patients with MDD had a different prevalence and intensity of pain compared to healthy controls [8]. MDD and pain share biological pathways and neurotransmitters, which suggests that simultaneous treatment of both conditions may be effective [9].

Our previous animal model studies have illustrated the therapeutic effects of EA against inflammatory pain via neuronal and nonneuronal pathways [10, 11]. EA stimulated secretion of endogenous opioids through the anesthesia pain descending pathway in the central neural system [12]. Furthermore, several clinical studies have indicated that EA relieves chronic pain [13], such as knee osteoarthritis [14], low back pain [15–17], neck pain [18, 19], and shoulder pain [20]. According to previous studies of depressed rats, the antidepressive efficacy of EA treatment has been achieved through the mechanisms of enhancing 5-HT synthesis [21], raising protein levels of phosphorylated extracellular regulated protein kinases [22], and suppressing inflammatory cytokines [23] in the hippocampus. Moreover, EA potentially regulates the dopaminergic synapse signaling pathway by activating reward system [24].

However, among patients with major depression, there were around 10%–30% who did not improve under the use of antidepressants [25]. In addition, selective serotonin reuptake inhibitors (SSRIs) often take weeks to exert their full effects in treating MDD [26] and leave most patients with considerable residual symptoms. Therefore, researchers have endeavored to discover other possible mechanisms that can be used in the treatment of depression. In particular, two noteworthy studies have discovered the rapid antidepressant action of ketamine [27, 28]. The level of beta form of calcium/calmodulin-dependent protein kinase type II (*β*CaMKII) was significantly upregulated in the lateral habenula of depressive animal models. The *β*CaMKII was one of the downstream factors controlled by NMDAR. By blocking the NMDAR-dependent burst firing of lateral habenula, Ketamine could reverse the suppression of reward center and caused rapid antidepressant effects.

On the basis of the aforementioned findings, we assumed that EA ameliorates pain and MDD. To further uncover the mechanisms linking EA, comorbidity, and NMDARs, we established a chronic pain and depression comorbidity model and used EA as the intervention. This study improves our understanding of how acupuncture treats comorbidities, using findings obtained through behavioral observation and the measurement of biomarkers in different brain cores.

## 2. Methods

### 2.1. Experimental Animals and Ethical Considerations

All animals were treated in accordance with the National Institute of Health Guide for the Care and Use of Laboratory Animals, and the study protocol was approved by the ethics committee of the China Medical University, Taichung, Taiwan (permit no. 2017-374). C57/B6 male mice weighing approximately 22–25 g and aged 8–12 weeks were purchased from the BioLASCO Animal Center, Taipei, Taiwan. Animals were housed in plexiglass cages in a temperature-controlled room (25 ± 2°C) with a relative humidity of 60 ± 5% and were fed a diet of standard rat chow and water ad libitum.

### 2.2. Chronic Inflammatory Pain and Depression Model

There were four groups in the present study. A total of eight mice in each group were determined as the minimum number necessary to carry out the experiments. All experiments were performed at laboratory during daylight hours. C57/B6 mice were randomly (simple random sampling) divided into four groups and then anesthetized with 1% isoflurane for CFA injections and EA treatment. Then mice were injected with 20 *μ*l saline (pH 7.4, buffered with 20 mM HEPES) or CFA 20 *μ*l (complete Freund's adjuvant; 0.5 mg/ml heat-killed M. tuberculosis (Sigma, St. Louis, MO)) as previous study [29] in the plantar surface of the hind paw. CFA was used to induce intraplantar chronic inflammation. The 20 *μ*L of saline and 20 *μ*L of CFA were administered thrice: at baseline, at day 7, and at day 14. The four groups and their treatments were as follows: (1) control group: anesthesia with normal saline injection; (2) chronic inflammatory pain (CIP) group: anesthesia with CFA injections to induce chronic inflammatory pain and depression; (3) EA group: anesthesia with CFA injections and EA manipulation; and (4) sham EA group: anesthesia with CFA injections and sham EA to determine the roles played by acupoints.

### 2.3. Electroacupuncture and Sham Electroacupuncture

EA treatment was applied by stainless steel acupuncture needles (1.5″ inches, 30G, YU KUANG, Taiwan) that were inserted into the muscle layer at a depth of 3-4 mm at the ST36 acupoint. Several articles indicated the analgesic effect and pathway of EA on the ST36 acupoint [10, 11]. Electrical square pulses generated by the stimulator were delivered for 15 min—at 2 Hz, with 100 *μ*s between each pulse and a 1 mA amplitude. EA was carried out in the morning (9:00-10:00 am), twice a day, from day 14 to day 28. The same treatment was administered to a nonacupoint (3 mm away from ST36 but not GB34 [30, 31]) in the sham control group. By comparing the two groups, we aimed to demonstrate the specificity of acupoints under EA treatment. According to a review article [32], researchers proved that, through the different stimulation site with electroacupuncture or manual acupuncture, different afferent fibers such as thick myelinated A*α* and A*β*, thin myelinated A*δ*, and thinner unmyelinated C fibers would be stimulated with different treatment effects. We did not want to explore the different effects between EA and manual acupuncture in this study, so we did not establish a group with manual acupuncture on ST36.

### 2.4. Behavioral Tests for Chronic Pain: von Frey Test and Hargreaves Test

Beginning the first day after intraplantar CFA injection, mechanical sensitivities were tested every other day for 28 days. All experiments were performed at room temperature (approximately 25°C), and the stimuli were applied only when the animals were calm but not sleeping or grooming. Mechanical sensitivity was measured by testing the force of response to the stimulus from three applications of electronic von Frey filaments (North Coast Medical, Gilroy, CA, USA). The average thermal pain from three applications was measured using a Hargreaves test IITC analgesiometer (IITC Life Sciences, Woodland Hills, CA, USA).

### 2.5. Behavioral Tests of Depression: Forced Swimming Test and Open Field Test

EthoVision XT 8.5 (Noldus Information Technology, Wageningen, Netherlands) video-tracking software was used to automatically score anxiety-like behaviors in a forced swimming test (FST) and open field test (OFT) on day 28. The FST apparatus was a plastic cylinder (47 cm height, 38 cm inside diameter) containing 38 cm of water at 25 ± 1 °C. The water level was deep enough (18 cm) so the tail of the mouse never touched the bottom. The water was replaced between each test. The mice were exposed to forced swimming and their immobile behavior is measured and considered a “depression-like” phenotype. The FST comprised two phases. In the initial 15 min habituation session, which, as a training procedure, was excluded from the data analysis, the mice were individually forced to swim in a plastic cylinder. If an animal appeared to be in serious distress like tiredness or floating disability, the animal would be removed from the water and excluded from the experiment. After a period of vigorous swimming, all mice economized their movements to only those necessary to maintain their head above the water, with no other displacement. The 5 min test sessions began 24 hours later. The duration of immobilization was measured. After the test, the mice were removed and dried with a towel before being returned to their home cages. Increased immobility in the forced swimming test was indicative of depression-like behavior.

The OFT box was composed of black acrylic plastic that formed a 30 × 30 cm square with a wall height of 15 cm. The box was divided into nine equal squares. Each mouse was placed in the center zone of an open field at the beginning and allowed to explore the maze for 15 min. The distance of crossing the central zone, the duration in the center area, and the total movement in the open field were analyzed. A low frequency of crossing the central zone or a short duration of time spent within the central zone was considered a validation of depression.

### 2.6. Tissue Sampling and Western Blot

The mice were sacrificed using CO_2_ to minimize their suffering. The prefrontal cortex (PFC), hippocampus, and hypothalamus were harvested on day 28 and then immediately excised to extract proteins. Total proteins were prepared in lysis buffer containing 50 mM Tris-HCl pH 7.4, 250 mM NaCl, 1% NP-40, 5 mM EDTA, 50 mM NaF, 1 mM Na3VO4, 0.02% NaN_3_, and 1x protease inhibitor cocktail (AMRESCO). The extracted proteins (30 *μ*g per sample assessed through BCA protein assay) were subjected to 8% SDS-Tris glycine gel electrophoresis and transferred to a PVDF membrane. The membrane was blocked with 5% nonfat milk in TBS-T buffer (10 mM Tris pH 7.5, 100 mM NaCl, 0.1% Tween 20), incubated with first antibody (anti-pNR1, anti-NR1, anti-pNR2B, anti-NR2B, and anti-pCaMKII*α*) in TBS-T with 1% bovine serum albumin, and incubated for 1 hour at room temperature. Peroxidase-conjugated anti-rabbit antibody (1 : 5000) was used as a secondary antibody. The bands were visualized by an enhanced chemiluminescence substrate kit (PIERCE) with LAS-3000 Fujifilm (Fuji Photo Film Co. Ltd.). Wherever applicable, the image intensities of specific bands were quantified with NIH ImageJ software (Bethesda, MD, USA).

### 2.7. Immunofluorescence Staining

A total of 4 subjects, from the Con, CIP, EA, and sham EA groups, were anesthetized using 1% isoflurane through inhalation and intracardially perfused with saline followed by 4% paraformaldehyde. The brain was immediately dissected and postfixed with 4% paraformaldehyde at 4°C overnight. The postfixed tissues were placed overnight in 30% sucrose for cryoprotection at 4°C. The brain was embedded in OCT and instantaneously frozen using liquid nitrogen prior to storage at −80°C. The coronal sections containing the medial PFC (mPFC), hippocampal CA1, and hypothalamus were cut into 16 *μ*m thick slices through cryosectioning. The samples were incubated with blocking solution, which consisted of 3% bovine serum albumin (BSA), 0.1% Triton X-100, and 0.02% sodium azide, for 2 h at room temperature. Following blocking, the brain samples were incubated overnight with the primary antibodies, which was prepared in BSA solution at 4°C overnight. The secondary antibodies, peroxidase-conjugated anti-rabbit antibody, (1 : 5000) were used for incubation at room temperature for 2 h prior to fixation with cover slips for immunofluorescence visualization. Using a microscope (Olympus, BX-51, Japan), we searched for the presence of immune-positive neurons among the mPFC, hippocampal CA1, and hypothalamus slices.

### 2.8. Statistical Analysis

The experimental results for each group are expressed as means ± standard deviation. A paired *t*-test and one-way ANOVA were performed for intragroup and intergroup statistical analyses. The one-way ANOVA was followed by Tukey's post hoc test. Statistical significance was indicated if *p* < 0.05. SPSS for Windows (version 21.0, SPSS, Chicago, IL, USA) was used for all statistical analyses.

## 3. Results

### 3.1. Electroacupuncture Significantly Attenuated Mechanical and Thermal Hyperalgesia in the Chronic Pain and Depression Comorbidity Model

**Figure 1:**
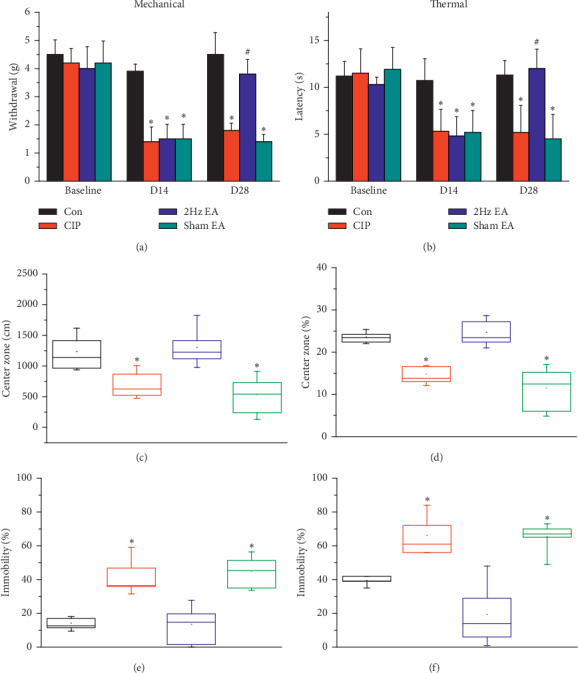
EA significantly attenuated mechanical, thermal hyperalgesia, and depression induced by CFA. (a) Changes in the withdraw threshold of mice in the von Frey test. (b) Changes in the withdraw latency of mice in the radial heat test. Figures 1(c) and 1(d) present the day 28 OFT results. After accepting EA, the time mice stayed in the center area longer. (c) The distance mice stayed in the central area. (d) The percentage of time mice spent in the center area. Day 28 FST results are presented in Figures 1(e) and 1(f). EA reduced the immobility of mice. (e) Duration of immobility spent in the FST. (f) Frequency of immobility counted in the FST. ^*∗*^*p* < 0.05 for CIP versus control. ^#^*p* < 0.05 for EA versus CIP.

As shown in Figure 1(a), mechanical sensitivity did not differ among the four groups under basal conditions. A significant lower pain threshold, namely, mechanical hyperalgesia, was observed in the CIP group and sham EA group on days 14 and 28 when compared with the control group (Figure 1(a), *p* < 0.05, *n* = 8). However, EA significantly reduced mechanical hyperalgesia (Figure 1(a), *p* < 0.05, *n* = 8).  Figure 1(b) showed similar results. There was no significant difference of withdrawal latencies among all groups prior to CFA injections. However, on days 14 and 28, the withdrawal latencies in the CIP group and the sham EA group (Figure 1(b), *p* < 0.05, *n* = 8) were shorter than that among the control group (Figure 1(b), *p* < 0.05, *n* = 8). Interestingly, EA also significantly reduced thermal hyperalgesia (Figure 1(b), *p* < 0.05, *n* = 8).

### 3.2. Electroacupuncture Significantly Attenuated Depressive Behavior in the Chronic Pain and Depression Comorbidity Model

On day 28, the results of the OFTs were presented in Figures 1(c) and 1(d). Mice among the four groups performed similarly in the total distance of the OFT. The distance in the central area was significantly shorter in the CIP group and sham EA group in comparison to that of the control group (Figure 1(c), *p* < 0.05, *n* = 8). EA significantly increased the distance in the central area (Figure 1(c), *p* < 0.05, *n* = 8). The percentage of time spent in the center of the OFT, depicted in Figure 1(d), showed similar result as Figure 1(c). The results of FST on day 28 were shown as Figures 1(e) and 1(f). Among CIP group and sham EA group, the percentage of immobile time and the frequency of counted immobility were higher than control group. EA reduced the immobility of mice.

### 3.3. Electroacupuncture Upregulated Reduced NMDARs in CIP Mouse Brain

To test the effect of CIP on NMDA levels, we investigated the effect of EA as exerted through the NMDA signaling pathway in the mice prefrontal cortex (PFC) region. Expression of pNR1 was significantly decreased in the PFC of CIP mice (Figure 3(a), *p* < 0.05, *n* = 6), and the phenomenon was significantly reversed by EA treatment (Figure 3(a), *p* < 0.05, *n* = 6). In addition, pNR1 expression was significantly reduced in the sham EA group (Figure 3(a), p ˂ 0.05, *n* = 6). Similar results were also obtained in NR1 protein levels (Figure 3(b), *n* = 6). Furthermore, pNR2B were significantly decreased in the CIP group compared to the control group (Figure 3(c), *p* < 0.05, *n* = 6). In contrast, this decrease was significantly reversed by EA treatment (Figure 3(c), *p* < 0.05, *n* = 6). Sham EA operation did not have effect on decreased pNR2B protein levels (Figure 3(c), *p* < 0.05, *n* = 6). Notably, a similar tendency was observed in the NR2B protein expression (Figure 3(d), *n* = 6). Moving along the signaling cascade, we next test the protein levels of pCaMKII*α*. Accordingly, we indicated a significantly reduced expression of pCaMKII*α* in CIP group (Figure 3(e), *p* < 0.05, *n* = 6). The pCaMKII*α* protein levels were significantly increased in the EA group (Figure 3(e), *p* < 0.05, *n* = 6) but not in the sham EA group (Figure 3(e), *p* < 0.05, *n* = 6). The aforementioned results indicate that the NMDA signaling pathways are commonly associated with the chronic pain and depression comorbidity mice.

We next examined the NMDA signal pathways in the mice hippocampus. It was ascertained that CIP induced a significant reduction of pNR1 in the CIP mice (Figure 4(a), *p* < 0.05, *n* = 6). EA has a significant increase in the pNR1 levels (Figure 4(a), *p* < 0.05, *n* = 6), which was not observed in the sham EA group (Figure 4(a), *p* < 0.05, *n* = 6). Similar tendency was obtained in the NR1 protein levels (Figure 4(b), *n* = 6). Again, the decrease of pNR2B and NR2B suggested a similar tendency as observed in mice hippocampus, displaying decreased protein levels in CIP mice (Figure 4(c) and4(d), *p* < 0.05, *n* = 6). In addition, the phenomena were rescued by EA treatment but not sham EA group (Figure 4(c) and 4(d), *p* < 0.05, *n* = 6). The pCaMKII*α* has lowered expressed in the CIP group (Figure 4(e), *p* < 0.05, *n* = 6) and then rescued by EA treatment but not sham EA group (Figure 4(e), *p* < 0.05, *n* = 6). Similar tendency was also observed in the mice hypothalamus (Figure 5, *n* = 6).

**Figure 2:**
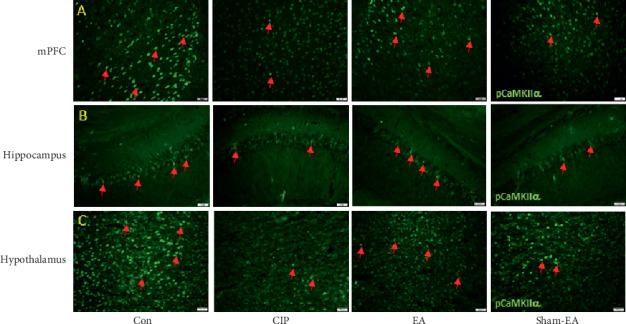
Expression levels of pCaMKII*α* in the mPFC, hippocampal CA1, and hypothalamus for all four groups. (a) Representative immunofluorescence staining of pCaMKII*α* (green) in the mice mPFC; (b) representative immunofluorescence staining of pCaMKII*α* (green) in the mice hippocampal CA1; (c) representative immunofluorescence staining of pCaMKII*α* (green) in the mice hypothalamus. Arrows indicate immunopositive neurons.

Furthermore, we utilized the immunofluorescence technique to identify the pCaMKII*α* protein expression in the mPFC, hippocampal CA1, and hypothalamus that is crucial for chronic pain and depression. The images of the mPFC area displayed significantly decreased pCaMKII*α* in the CIP and sham EA groups. These protein levels significantly increased in the EA group (Figure 2(a)). Additionally, the proteins pCaMKII*α* displayed similar tendencies in hippocampal CA1 and hypothalamus areas (Figures 2(b) and 2(c)).

## 4. Discussion

Mechanical and thermal hyperalgesia confirmed that CFA injections successfully evoked inflammatory pain. On day 28, the decreased distance traversed in the center square during the OFT and increased immobility during the FST demonstrated our successful induction of depression. As indicated by the difference between the CIP and EA groups, EA from days 14 to 28 reduced pain and depression. We also demonstrated the specificity of acupoint ST36 for the amelioration of comorbidity, by comparing data from the EA group and sham EA group.

Depression and pain affect each other. They also share neural circuitry and molecular signaling pathways; however, the same neuromolecular mechanisms sometimes lead to contrary effects in the different brain cores [9, 33]. For example, high-frequency repetitive transcranial magnetic stimulation (rTMS) over the left dorsolateral prefrontal cortex is an FDA approved therapeutic intervention for drug-resistant major depression [34]. The increasing levels of glutamate were found among responders accepting rTMS [35]. By contrast, glutamatergic excitatory neurotransmission in the lateral habenula causes depression [36, 37] by inhibiting the reward center involving regions such as the dopaminergic ventral tegmental area and serotonergic dorsal raphe nucleus [27, 28].

A study reported decreased expression of the NMDAR subunits NR2A (by −54%) and NR2B (by −48%) in the anterior PFC of people with depression [38]. Magnetic resonance spectroscopy also revealed lower glutamate/glutamine levels in the PFC [39]. Moreover, a study noted that animals with depression had (1) reduced levels of NMDAR subunits NR1 and NR2A in the hippocampus and (2) reduced NR1 in the PFC [40]. In particular, hippocampal volume reduction is the most replicated finding in neuroimaging studies of MDD [41]. Hypothalamic-pituitary-adrenal (HPA) axis hyperactivity and hypoactivity have been reported in depression, but using HPA activity as a depression biomarker is challenging [42]. Before the development of chronic pain, the HPA axis may become hyperactive; however, long-term hyperactivity may result in an exhausted stress system and ultimately a hypoactive HPA axis [43]. Therefore, we (1) chose the NMDAR subtype of the excitatory glutamate receptor family as the main biomarker to assess the antidepressive effect of EA and (2) chose the PFC, hippocampus, and hypothalamus as the main cerebral cores to study.

**Figure 3:**
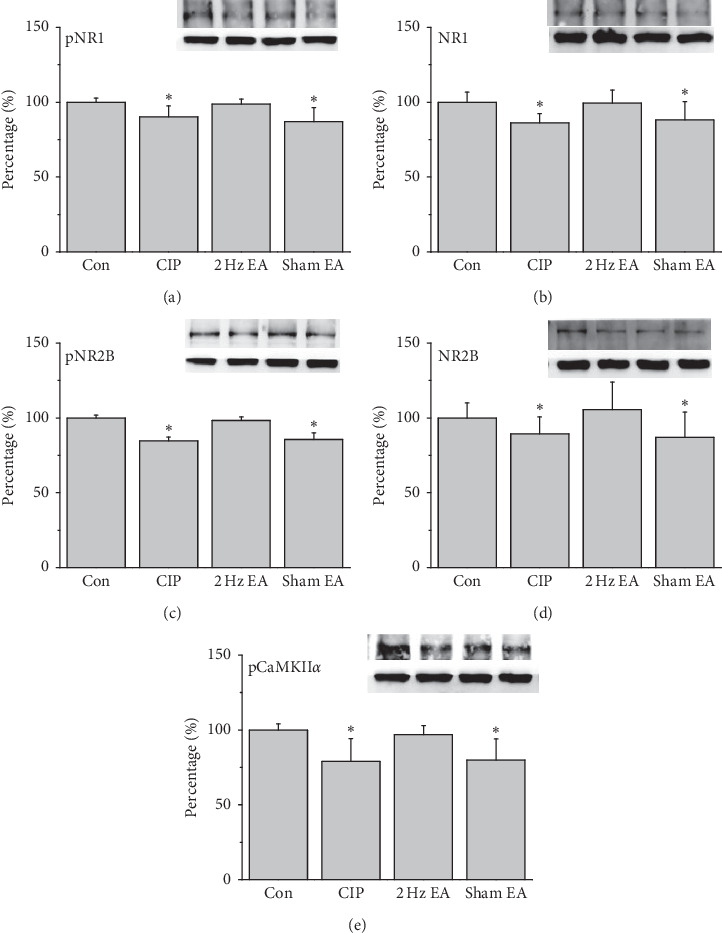
Protein levels of (a) pNR1 (b) NR1, (c) pNR2B, (d) NR2B, and (e) pCaMKII*α*, measured through Western blotting in mice mPFC. ^*∗*^*p* < 0.05 versus control. The Western blot bands at the top indicate the cropped target protein. The lower bands indicate the cropped internal controls (*β*-actin or *α*-tubulin).

**Figure 4:**
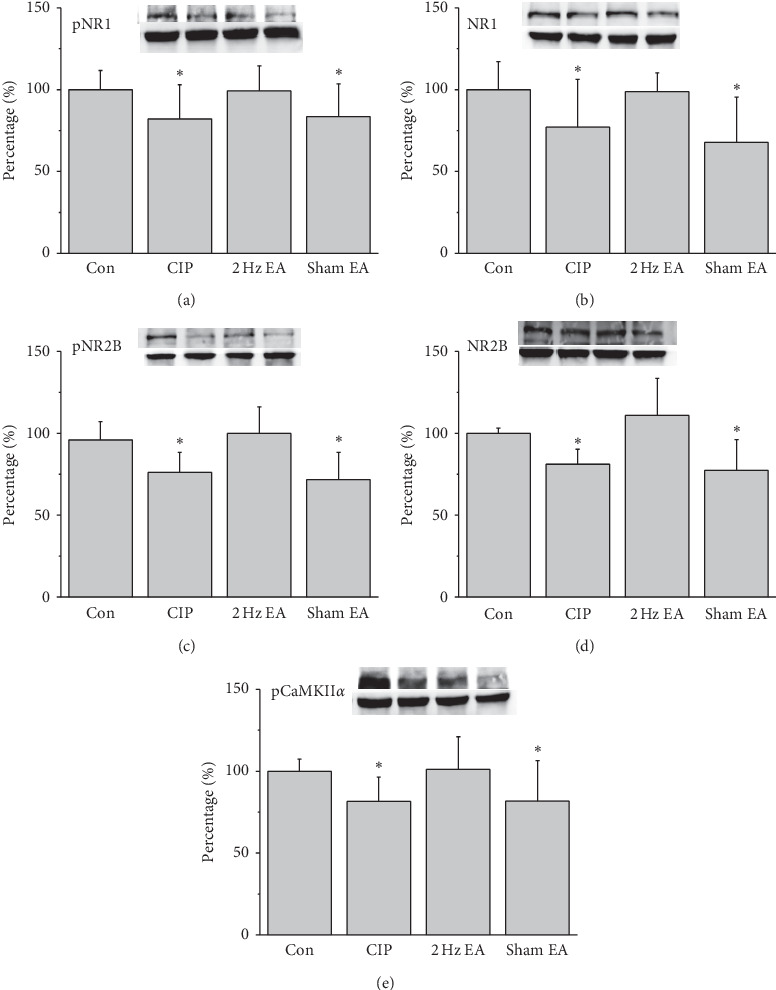
Protein levels of (a) pNR1 (b) NR1, (c) pNR2B, (d) NR2B, and (e) pCaMKII*α*, measured using Western blotting, in mice hippocampus. ^*∗*^*p* < 0.05 versus control. Western blot bands at the top indicate the cropped target protein. Lower bands indicate the cropped internal controls (*β*-actin or *α*-tubulin).

**Figure 5:**
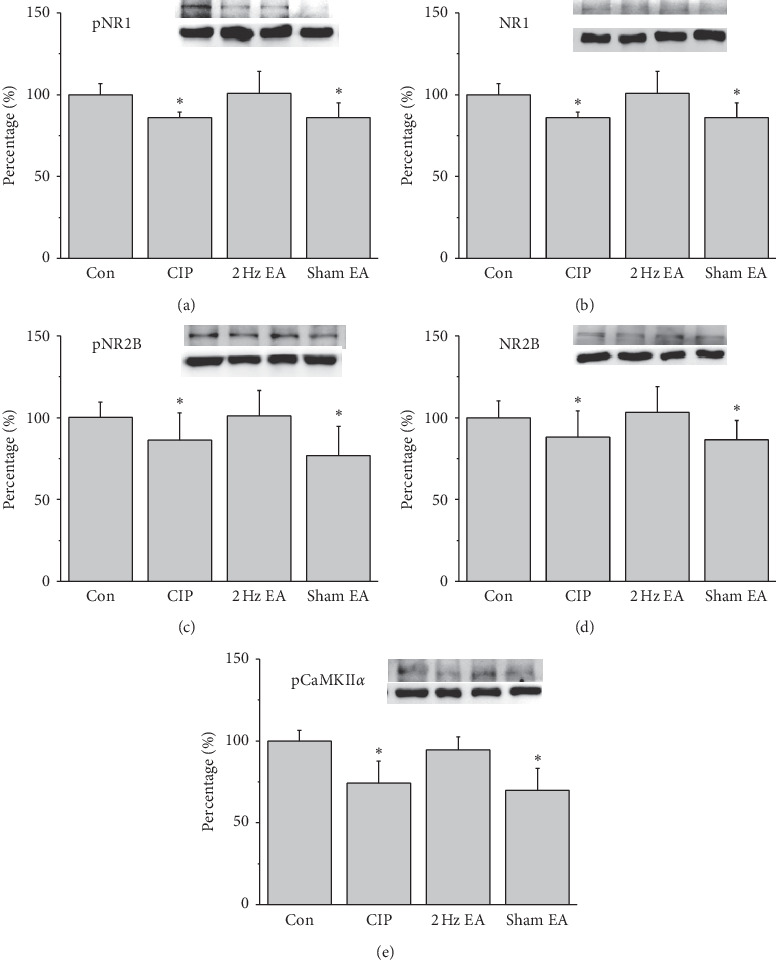
Protein levels of (a) pNR1 (b) NR1, (c) pNR2B, (d) NR2B, and (e) pCaMKII*α*, measured using Western blotting in mice hypothalamus. ^*∗*^*p* < 0.05 versus control. Western blot bands at the top indicate the cropped target protein. Lower bands indicate the cropped internal controls (*β*-actin or *α*-tubulin).

In the CIP and sham EA groups, the levels of pNR1, NR1, pNR2B, NR2B, and pCaMKII*α* decreased by day 28 in the PFC, hippocampus, and hypothalamus after three injections of CFA (Figures 3–5). The level of NMDARs among CIP and sham EA groups was lower than that of control group, with EA reversing the decreasing trends. In the same brain cores presented in Figure 2, our immunostaining data indicated that the protein levels pCaMKII*α*, which reflected potential downstream signaling mechanisms controlled by NMDA phosphorylation, were lower among CIP and Sham EA groups and reversed by EA. The antidepressive effect of EA could be partly disclosed by the present study, which showed EA reversing the decreasing glutamatergic excitatory neurotransmitters such as NMDARs and pCaMKII*α*. Our study has some limitations. First, we did not study cerebral areas such as the amygdala, cingulate cortex, and ventral tegmental area that are involved in comorbid chronic pain and depression. Some neurotransmitters, such as norepinephrine and dopamine, may also be affected by the comorbidity. Other antagonists of neurotransmitters should be investigated to understand EA's further mechanisms.

The current study provides evidence into how NMDA and related molecules participates in chronic pain and depression behaviors. EA significantly reversed decreases in NMDA and related molecules in the mouse PFC, hippocampus, and hypothalamus, suggesting involvement at multiple levels and emphasizing the possibility of leveraging them in functional therapeutic interventions. Clinical trials should be performed to clarity the subsequent application of TRPV1 as a treatment target for chronic pain and depression comorbidity.

## Data Availability

The data used to support the findings of this study are available from the corresponding author upon request.
